# The Detrimental Effects of No Trust: Active Decisions of No Trust Cause Stronger Affective and Behavioral Reactions Than Inactive Decisions

**DOI:** 10.3389/fpsyg.2021.643174

**Published:** 2021-07-07

**Authors:** Manon Schutter, Eric van Dijk, Erik W. de Kwaadsteniet, Wilco W. van Dijk

**Affiliations:** ^1^Social, Economic and Organisational Psychology, Leiden University, Leiden, Netherlands; ^2^Knowledge Centre Psychology and Economic Behaviour, Leiden, Netherlands

**Keywords:** trust, no trust, reciprocity, trust game, affect, behavior

## Abstract

In two experimental studies, we investigated the affective (Studies 1 and 2) and behavioral (Study 2) effects of not being trusted. In an adapted version of the Trust Game paradigm, participants were all assigned the position of Person B, and learned that their opponent (Person A) had decided to not let them divide monetary outcomes. This had either been an inactive decision (Person A had not offered them the option to distribute outcomes) or an active decision (Person A had taken away their option to distribute outcomes). Results of both studies reveal that reactions to not being trusted were significantly affected by whether this decision was active or inactive. Active decisions evoked a more negative evaluation toward Person A, led participants to experience more negative emotions, and lowered their satisfaction with the final outcome, even though payoffs and final earnings were held constant between the conditions (Study 1). In addition, when the decision not to trust had been an active decision, participants subsequently behaved less altruistic, as evidenced by significant lower allocations in a subsequent Dictator Game (Study 2). Interestingly, this reduction in altruism was not restricted to encounters with Person A, but also extended to an uninvolved other (Person C).

## Introduction

Trust is essential in many facets of our daily lives. This importance is evident in trust between family members or friends, but also in trust within economic settings. Consider, for example, online trading, which requires buyers to trust that upon payment the other party will indeed ship the ordered goods. In a similar vein, the concept of the sharing economy is becoming more and more popular, which entails individuals sharing their privately-owned goods (such as cars or even their houses) with complete strangers. For that, interpersonal trust is essential.

The importance of trust for societies has been widely acknowledged in the economic literature (e.g., Fukuyama, 1995; Knack and Keefer, 1997; Uslaner, 2000; Zak and Knack, 2001; Dincer and Uslaner, 2010). This literature not only reveals the benefits of trust in economic situations (such as economic growth), but also argues that low levels of trust lead to economic stagnation (such as lowered cooperation; e.g., Bijlsma-Frankema et al., 2015). Because of the impactful consequences of a lack of trust, it is not surprising that, besides the growing literature on trust, research on the topic of low trust and distrust is also expanding.

Trust has been defined as “a psychological state comprising the intention to accept vulnerability based upon positive expectations of the intentions or behaviors of another” (Rousseau et al., 1998, p. 395). People are not always prepared to accept such vulnerability. This means that at the behavioral level, people may be reluctant to interact with others they do not trust. For example, an eBay seller might be less willing to sell a product to a buyer with a lower reputation score, because the seller infers from this that the buyer is untrustworthy and may not pay for the product they would send. A decision not to engage in transactions may, of course, also be the result of the reluctance to take a risk; a consideration that, at the behavioral level, connects to the level of trust as the transaction itself may be considered riskier (see for example Bohnet and Zeckhauser, 2004; Eckel and Wilson, 2004). Not taking part in the transaction may be understandable from individual considerations like these, but the result may be suboptimal if it keeps people from making transactions. Under-trading and low levels of transactions may result in lower collective outcomes in which people miss out on the benefits of potential trade and transactions.

In the current paper we argue, however, that there may be more indirect negative effects of not trusting others than the more direct instrumental effects we discussed above. In addition to missing out on potential outcomes, there may also be affective consequences of not being trusted. How does it feel to realize that others do not trust you enough? Up until now, the empirical research on trust has not addressed this issue. However, learning about how people react to not being trusted is important if one wants to paint a comprehensive picture of the possible downsides of low levels of trust. Moreover, apart from evoking direct (negative) emotional reactions, we will show that not being trusted may also lead to new behavioral reactions which may shape subsequent interactions.

Sometimes people may explicitly communicate that they trust or do not trust you; for example, by verbally making such a statement. Oftentimes, however, we rely on other people’s decisions to make such inferences. As in the example above, a decision not to sell a product to you may not come with an explicit statement of the seller that she does not trust you. In experimental research, this behavioral focus is also apparent in the most widely used experimental game to study trust decisions and reactions: The Trust Game (TG).

This paradigm, initially coined the Investment Game (Berg et al., 1995), depicts a setting involving two persons. The first person (Person A; trustor) is endowed with a sum of money and decides whether to distribute this money him- or herself, or to send the money to an anonymous second person (Person B; trustee). If Person A decides to distribute the money him- or herself, both persons receive their allocated part and the game ends. However, if Person A decides to pass the money to Person B, the amount is tripled. Then, Person B makes the final decision concerning the distribution of the tripled sum of money. Within this paradigm, Person A’s decision to send the initial amount to Person B is interpreted as a behavioral measure of trust.

The general finding is that Persons B are quite willing to equally share the tripled outcomes with Person A. Such an act is interpreted as trustworthiness, but is also described as adhering to the norm of reciprocity (see e.g., Ostrom and Walker, 2003) and – relatedly – as a display of guilt aversion (e.g., Bellemare et al., 2019).

As we noted above, our key interest in the current paper is to assess how people affectively and behaviorally react when they learn that others did *not* show behavioral trust. In the TG this means assessing how B would react if A decides not to let B distribute the outcomes. It could be argued that if A decides to not let B distribute the outcomes, at the behavioral level there is nothing for B to reciprocate because there was no transaction to begin with. Indeed, if Person A in the TG decides to distribute the outcomes him- or herself, Person B has no behavioral option at all. It is therefore understandable that TG research did not (yet) address this issue; the game normally stops for Person B if A decides not to let B distribute outcomes.

To study affective and behavioral reactions we adjusted the traditional setup of the TG. In Study 1, we started by assessing B’s affective reactions to A’s decision not to let him/her distribute outcomes. In Study 2, we additionally studied behavioral reactions by offering B a behavioral option to respond to this decision. In both studies we furthermore investigated an important characteristic of how the decision not to let Person B divide outcomes was made; whether this was an active decision on A’s part or an inactive decision.

In the traditional TG, Person A decides whether to distribute outcomes him- or herself or to send the (then tripled) amount of money to Person B and leave the distribution to Person B. A’s decision to divide the outcomes oneself is then equivalent to *not offering* B the opportunity to show one’s trustworthiness. It seems plausible that Person B would react negatively toward this decision; he or she would probably prefer to be given the opportunity to allocate the outcomes. However, not offering an opportunity is not the only way in which a person can display his/her lack of trust. Another possibility is that a person actively *takes away* another’s opportunity to prove oneself trustworthy. Such a setting may, for example, resemble a team leader actively deciding to take away the decision power of one of the team members to make the decision him- or herself. Now, this team member started out having the chance to prove him- or herself trustworthy, but this chance was then taken from him or her (which we refer to as an active decision not to trust).

The issue of active versus inactive decisions connects to a broader body of research within the decision-making literature which studies reactions toward actions vs. inactions, also described as commissions vs. omissions (e.g., Kahneman and Tversky, 1982; Kahneman and Miller, 1986; Ritov and Baron, 1990, 1992; Spranca et al., 1991). Omissions describe decisions of inaction (e.g., not helping someone who is hurt), whereas commissions usually involve more active participation (e.g., hurting someone). Although both acts may result in the same outcome (e.g., someone ends up being hurt), active decisions are generally evaluated more negatively (in the case of adverse outcomes). In general, adverse outcomes resulting from harmful commissions seem to be more impactful than outcomes of harmful omissions (e.g., Ritov and Baron, 1990, 1992; Spranca et al., 1991). Spranca et al. (1991), for example, found that harmful commissions are considered to be worse decisions than harmful omissions. Likewise, research on actions and inactions, which are comparable to (active) commissions and (inactive) omissions, provides evidence for stronger affective as well behavioral responses as a result of action (e.g., Kahneman and Tversky, 1982; Kahneman and Miller, 1986). The issue of active vs. inactive decisions also connects to previous research on dictator games. When allocating money, taking money from others is viewed more negatively than not giving money to others (Krupka and Weber, 2013; Capraro and Vanzo, 2019).

Based on these findings, we expect that taking away an opportunity to prove one’s trustworthiness (i.e., an active decision not to trust) will engender stronger affective and behavioral reactions compared to merely not offering the chance to prove one’s trustworthiness (i.e., an inactive decision not to trust).

## Study 1: Affective Reactions Toward a No Trust Decision

In this first study, participants were presented with a trust game in which they as Person B learned that the decision of Person A not to let them distribute the outcomes had either been an active or an inactive decision. In the inactive condition, a decision not to trust the participant was modeled in the way it is usually depicted in trust games: Person A was endowed with money and decided not to let Person B distribute the (then tripled) outcomes. In the active condition, Person B was endowed with money, but A decided to take away this decision authority.

Researchers repeatedly found that affect and emotions are important antecedents of trust. For example, that affective states help people to make trust decisions (Jones and George, 1998), may influence the level of trust people have in others (Williams, 2001), and emotions, even unrelated to the situation of trust, affect people’s willingness to trust (Dunn and Schweitzer, 2005). The current setup allowed us to assess how people respond affectively to an active vs. inactive decision of no trust. Based on previous findings in research on action/inaction as well as on (no) trust, we expected that our participants would react negatively when finding out that Person A had decided to not let them distribute the outcomes, and that these reactions would be more negative when knowing that this had been an active decision as compared to when it had been an inactive decision.

At this point it is important to note that participants did not receive any explicit statement from Person A. A did not inform them that he/she did not trust them; all they knew was that A had decided to not let them divide the outcomes, but instead decided to allocate the outcomes him- or herself. In TG research this is generally interpreted as a no-trust decision, but it is conceivable that reactions are a mixture of a feeling of not being trusted but possibly also to the potential reduction of collective outcomes that go with it (after all, outcomes are not tripled anymore), and possibly perceptions of one’s opponent being competitive or self-interested. In the current study, we therefore not only asked participants whether they felt their opponent did not trust them, but we also assessed these alternative responses.

## Materials and Methods

### Participants and Design

An a priori power analysis (using G^∗^Power3, Faul et al., 2007) was conducted to indicate the required sample size (for one-way ANOVAs fixed effects, two-tailed; alpha = 0.05; power = 0.80). In total, a sample size of 128 was needed in order to detect a medium effect size (*f* = 0.25; Cohen, 1969). We collected data of 133 participants, but data from nine participants were excluded because they had recently participated in a highly similar study. *Post hoc* analysis revealed that with our sample we obtained a power level of 0.79.

Participants in this study were 124 students (90 women, 34 men; *M* = 20.2 years, *SD* = 2.28)^1^ from Leiden University who were paid 3 euros for participation. Study 1 had a two conditions (type of decision: inactive vs. active) between participants design. This study involving human participants was reviewed and approved by the Psychology Research Ethics Committee of Leiden University. The participants provided their written informed consent to participate in this study. The original data of Study 1 are publicly available and accessible via: https://osf.io/3jw6p/?view_only=bc512df8e91f4ac79d7a59f6f4bb61b8.

### Procedure

Participants were invited to the laboratory at the Faculty of Social Sciences at Leiden University for a study on decision making. They were all seated in separate cubicles each containing a computer that guided them through the experiment.

Participants were randomly assigned to either the ‘inactive condition’ or the ‘active condition.’ They learned that they would take part in an interaction with an anonymous other and that we would refer to them as Person A and Person B; this was maintained throughout the study (e.g., we did not use labels such as ‘opponent’). All participants were assigned to the role of Person B and full anonymity during and after the study was ensured. Unknown to the participants, the decision of Person A was preprogrammed as we were merely interested in Person B’s reactions to a no trust decision.

Depending upon condition, participants were introduced to either the traditional TG (inactive condition) or the modified TG (active condition). In the inactive condition, participants learned that Person A had received 20 chips (worth €0, 10 each) and had the following options: (Option 1) to divide the 20 chips him- or herself and therefore, the number of chips to divide would remain the same, or (Option 2) to pass all chips to Person B (i.e., the participant) and provide this person with the opportunity to divide the then tripled amount of 60 chips. In the active condition, Person B – instead of Person A – started out having 20 chips (worth €0, 10 each). Here too, Person A had two options: (Option 1) take this amount from Person B and thereafter divide the 20 chips him- or herself, or (Option 2) leave the initial 20 chips with Person B and give him or her the opportunity to thereafter divide the then tripled amount of 60 chips. The outcome structure and decision process were thus identical in both conditions.

After an explanation of the procedure, participants filled in three comprehension checks (e.g., ‘Which role are you assigned to?’; all between 95.2 and 100% correctly answered; see Supplementary Materials for the results). Comprehension of the procedure was assured by revealing the right answer after each check. After a simulated decision time of 45 s, participants (as being Person B) learned that Person A had opted for Option 1. In the inactive condition this meant that Person A would allocate the initially received amount of money him- or herself (i.e., the opportunity to distribute the money was not provided to B). In the active condition this meant that Person A took the initial amount from B to allocate this him- or herself (i.e., took the opportunity to distribute the money away from B). We always described the mentioned options avoiding words such as ‘trust’ and ‘distrust.’

Participants were then informed that Person A was thinking about the final division of the 20 chips, and that meanwhile we wanted them to fill out a series of questions. Participants were thus not informed about how many chips A allocated to them while evaluating A’s decision not to let them distribute outcomes. Unless reported differently, all items were measured on seven-point scales by which participants could indicate their level of agreement (1 = *absolutely disagree*; 7 = *absolutely agree*). The following four questionnaires were first administered: evaluations of Person A’s decision (e.g., ‘kind’), experienced emotions (e.g., ‘sad’), affective reactions (e.g., ‘rejected’), and the perceived main motives of Person A (e.g., ‘Person A did not have trust in me providing him/her a fair number of chips’ and ‘Person A distrusted me’). See Tables 1–4 for all measured items per construct.

**TABLE 1:** Factor loadings based on an exploratory factor analysis with a principal axis factoring and direct oblimin rotation on 12 evaluation items (*N* = 124).

	1. General negative evaluation	2. Perceived expectedness	Communality
9. Unkind	0.80		0.66
12. Selfish	0.75		0.57
4. Unfair	0.70		0.45
5. Self-centered	0.69		0.50
6. Kind	−063		0.39
10. Immoral	0.61		0.49
2. Unjustified	0.56		0.35
8. Cooperative	−0.56		0.36
3. Justified	−0.50		0.23
7. Unexpected		−0.97	0.94
11. Expected		0.88	0.79
1. Surprising		−0.85	0.74
Percentage of variance	37.22	16.80	
Eigenvalue	4.92	2.28	
Cronbach’s α	0.87	0.93	

*Factor loadings < 0.30 are suppressed.*

**TABLE 2:** Factor loadings based on an exploratory factor analysis with a principal axis factoring and direct oblimin rotation on 15 items measuring emotions and affective reactions (*N* = 124).

	1. Negative emotions	2. Positive emotions	3. Personal rejection	Communality
2. Angry	0.78			0.57
9. Frustrated	0.72			0.62
11. Annoyed	0.72			0.64
14. Sad	0.66			0.44
12. Disappointed	0.60			0.61
3. Excluded	0.50			0.44
15. Rejected	0.41		0.38	0.46
4. Happy		0.81		0.70
13. Satisfied		0.79		0.65
10. Appreciated		0.63		0.46
8. Involved		0.61		0.42
1. Grateful		0.60		0.39
6. Distrusted			0.68	0.58
5. Upset	.32		0.53	0.50
7. Powerless			0.43	0.33
Percentage of variance	32.23	15.02	4.55	
Eigenvalue	5.31	2.73	1.16	
Cronbach’s α	0.87	0.82	0.67	

*Factor loadings < 0.30 are suppressed. Items that cross loaded on more than one factor were assigned to the factor on which they loaded the highest.*

**TABLE 3:** Factor loadings based on an exploratory factor analysis with a principal axis factoring and direct oblimin rotation on eight items measuring the perceived underlying motives (*N* = 124).

	1. Low trust/risk	2. Control	Communality
4. Person A did not have trust in me providing him/her a fair number of chips	0.74		0.67
3. Person A distrusted me	0.66		0.47
6. Eventually, Person A will keep all the chips to him- or herself	0.50		
1. Person A did not want me to possess any chips	0.47		0.21
5. Person A has a competitive mindset	0.33		0.23
7. Person A did not want to be dependent upon my division of the chips		0.79	0.58
8. Person A wanted to keep control over the decision		0.75	0.58
2. Person A found it too risky to let me divide the chips	0.43		0.85
Percentage of variance	32.60	9.83	
Eigenvalue	3.05	1.18	
Cronbach’s α/Pearson’s *r*	0.69	0.57	

*Factor loadings < 0.30 are suppressed.*

**TABLE 4:** Factor loadings based on an exploratory factor analysis with a principal axis factoring and direct oblimin rotation on eight items measuring behavioral intentions (*N* = 124).

	1. Negative behavioral intentions	Communality
1. I want to keep as much distance between us as possible	0.87	0.83
5. I want to cut off the relationship with the other person	0.83	0.86
3. I want to avoid the other person	0.81	0.66
6. I find it difficult to act warmly toward the other person	0.75	0.58
4. I want to retribute against the other person	0.52	0.30
2. I want to prove that I am a trustworthy person		0.06
Percentage of variance	48.89	
Eigenvalue	3.24	
Cronbach’s α	0.85	

*Factor loadings < 0.30 are suppressed.*

For a more comprehensive view, we also added some exploratory measures. First, blaming Person A (‘I blame Person A’), because of a higher perceived responsibility of the other party after actions compared to inactions (Spranca et al., 1991). Furthermore, to explore whether the action-inaction manipulation might also have behavioral effects, we assessed behavioral intentions toward Person A. We asked participants about how they expected themselves to behave in a future interaction toward the same Person A they had just interacted with. Items were derived from the Transgression-Related Interpersonal Motivations Scale, TRIM (McCullough et al., 1998; e.g., ‘I want to retribute against the other person’). And third, we assessed what allocation they expected from A (‘Indicate how you expect Person A to divide the 20 chips’). After filling out all items, participants were informed that Person A had distributed the 20 chips evenly (i.e., 10 chips to A, 10 chips to B), and we asked them about their satisfaction with this final outcome (see Supplementary Materials).

Manipulation checks were administered to examine whether the manipulation of the type of decision (active vs. inactive) had been successful (e.g., ‘What was the interaction’s starting point: Person A vs. Person B received 20 chips?’). We also briefly explained the difference between actions and inactions, and asked participants to indicate to what extent they thought Person A’s choice for Option 1 was inactive vs. active. Five comprehension questions were asked to check whether the participants had understood the main characteristics of the TG (e.g., ‘Which role are you assigned to?’; all between 90.3 and 100% correctly answered).

Finally, participants were debriefed, paid (plus €1 extra for the 10 chips they earned) and thanked for their participation.

## Results

### Manipulation Check

In the inactive condition, all participants (100%) correctly answered that Person A started out with 20 chips. In the active condition, almost all participants (90.3%) correctly answered that Person B started out with 20 chips. Furthermore, participants rated Person A’s decision to distribute the outcomes him- or herself as significantly more active in the active condition (*M* = 5.47, *SD* = 1.85) than in the inactive condition (*M* = 3.32, *SD* = 2.27), *t*(117.35) = −5.77, *p* < 0.001. These findings indicate that our manipulation of type of decision (active vs. inactive) was perceived as intended.

### Exploratory Factor Analyses (EFAs)

On each measured construct, separate exploratory factor analyses (EFAs) were conducted to explore the structure of the data within the measured constructs. All EFAs were conducted using principal-axis factor extraction and oblique rotation (direct oblimin rotation). Each sample of data appeared suitable for factoring, as indicated by the high strength of the relationships among variables (Kaiser–Meyer–Olkin, KMO = between 0.71 and 0.85, all above the recommended 0.60), and all significant Bartlett’s tests of sphericity (all *p*s < 0.001). See Supplementary Materials for more details.

Results of the EFAs narrowed the measured items down to the following factors per measured construct. First, evaluations of Person A’s decision were narrowed down to two factors: ‘General negative evaluation’ (nine items; Cronbach’s α = 0.87), and ‘perceived expectedness’ (three items; α = 0.93). Second, emotions and affective reactions following Person A’s decision were narrowed down to three factors: ‘Negative emotions’ (seven items; α = 0.87), ‘positive emotions’ (five items; α = 0.82), and ‘personal rejection’ (three items; α = 0.67). Third, the perceived main motives underlying Person A’s decision (eight items) were narrowed down to two factors: ‘Low trust/risk’ (six items; α = 0.69), and ‘control’ (two items; Pearson’s *r* = 0.57, *n* = 122, *p* < 0.001). See Tables 1–3 for all items per factor and corresponding factor loadings.

As an additional measure, behavioral intentions toward the not trusting person (Person A) were narrowed down to one factor and one single item: ‘Negative behavioral intention’ (five items; α = 0.85) and the item ‘I want to prove that I am trustworthy.’ See Table 4 for all items per factor and corresponding factor loadings.

### Dependent Variables

Unless reported differently, the difference between the means of our two conditions (active vs. inactive decision) on the dependent variables were analyzed using separate one-way Analyses of Variance (ANOVAs).

#### Evaluations of Person A’s Decision

A significant effect of the type of decision was found on participants’ ‘general negative evaluation’ toward Person A’s decision, *F*(1,122) = 6.17, *p* = 0.01, $\eta_{\text{p}}^{2}$ = 0.05. Participants indicated to experience a stronger negative evaluation toward A in the active condition (*M* = 4.68, *SD* = 1.03) than in the inactive condition (*M* = 4.23, *SD* = 1.00).

A marginal significant effect was found of the type of decision on the ‘perceived expectedness’ of A’s decision, *F*(1,122) = 9.88, *p* = 0.09, $\eta_{\text{p}}^{2}$ = 0.02. After an active decision, participants tended to find Person A’s decision less expected (*M* = 3.94, *SD* = 1.76) compared to after an inactive decision (*M* = 4.51, *SD* = 1.87).

#### Emotions and Affective Reactions

Participants experienced more ‘negative emotions’ in the active condition (*M* = 3.82, *SD* = 1.28) than in the inactive condition (*M* = 3.28, *SD* = 1.24), *F*(1,122) = 5.68, *p* = 0.02, $\eta_{\text{p}}^{2}$ = 0.05.

Both ‘positive emotions’ (overall *M* = 2.30, *SD* = 0.87, *p* = 0.97) and ‘personal rejection’ (overall *M* = 4.35, *SD* = 1.24, *p* = 0.62) did not reveal significant differences.

#### Perceived Main Motives

The motive of ‘low trust/risk’ was rated as more applicable to A’s decision in the active condition (*M* = 5.35, *SD* = 0.82) than in the inactive condition (*M* = 5.00, *SD* = 0.99), *F*(1,122) = 4.58, *p* = 0.03, $\eta_{\text{p}}^{2}$ = 0.04.

No significant difference was found on ‘control’ as a perceived main motive for Person A (overall *M* = 6.34, *SD* = 0.78, *p* = 0.73).

### Additional Measures

#### Blame

Participants in the active condition ‘blamed’ Person A to a significant higher extent for the outcome (*M* = 4.13, *SD* = 1.89) than participants in the inactive condition (*M* = 3.16, *SD* = 1.78), *F*(1,122) = 8.65, *p* = 0.004, $\eta_{\text{p}}^{2}$ = 0.07.

#### Behavioral Intentions

Participants indicated how they would react if they encountered Person A in a future interaction. The active and inactive condition did not differ significantly on their expected ‘negative behavioral intention’ toward Person A (*p* = 0.28, overall *M* = 2.52, *SD* = 1.18). Neither did these groups differently indicate that they would ‘prove themselves trustworthy toward A’ in a subsequent interaction (*p* = 0.95, overall *M* = 5.12, *SD* = 1.47).

#### Expectations of A’s Decisions

Participants in the inactive condition indicated that they had already expected Person A to choose Option 1 (*M* = 4.65, *SD* = 1.91) and more so than participants in the active condition (*M* = 3.76, *SD* = 2.07), *F*(1,122) = 6.16, *p* = 0.01, $\eta_{\text{p}}^{2}$ = 0.05.

Participants were also asked to indicate how many chips they expected Person A to allocate to themselves (A) vs. the participant (B). We analyzed the expected number of chips allocated to Person A. One participant was excluded from this analysis as the expected allocations to A and B did not sum up to 20 chips. Both participants in the active condition (*M* = 15.48, *SD* = 3.90) and inactive condition (*M* = 14.68, *SD* = 3.67) expected Person A to keep approximately 75% of the available 20 chips to themselves, *F*(1,121) = 1.36, *p* = 0.25, $\eta_{\text{p}}^{2}$ = 0.01.

## Discussion

Results of our first study reveal that affective reactions to not being offered the chance to distribute outcomes are contingent on whether this had been caused by an active or an inactive decision. Participants evaluated Person A’s decision more negatively when this was an active decision. Relatedly, they held a stronger negative evaluation toward A, blamed Person A to a higher extent for the choice made, and – although the payoffs were held constant – were less satisfied with the final outcome. As we noted earlier, participants did not receive an explicit message from A that he/she did not trust them, but only learned what A had decided. Therefore, to check whether participants indeed interpreted this decision as a lack of trust, we explicitly asked them to what extent they felt that A’s decision was motivated by low trust in them. As expected, participants felt that an active decision was more indicative of a low level of trust than an inactive decision. We will return to this observation in the Section “General Discussion.”

In addition to the affective responses, we also explored behavioral intentions toward the Person A. In this study, the type of decision A had made (active vs. inactive) did not influence behavioral intentions. However, it should be noted that these were only self-reported expectations about how they would respond, had our participants been given an opportunity to do so. As such, one might wonder how they would actually behave in subsequent encounters. We therefore ran Study 2 to study actual behavior.

A notable finding of Study 1 is that participants in both conditions expected Person A to unevenly divide the chips in his/her own advantage; they expected A to keep around 75% of the available chips. These expectations of being disadvantaged could have negatively affected their judgments about Person A and might have also affected their (expected) behavioral responses toward A. In other words, their reactions could be influenced by a mixture of feeling not trusted and expecting to be disadvantaged. To prevent this expectation of influencing their affective and behavioral reactions, in Study 2 we used a setting in which participants again learned that A had decided to allocate outcomes him- or herself, but immediately afterward also learned that A divided the money evenly. Introducing this fixed and equal final division in Study 2 allowed us to study affective and behavioral reactions, disentangled from anticipated or perceived disadvantage.

## Study 2: Affective and Behavioral Reactions to a No Trust Decision

In Study 1, we assessed affective reactions of participants when finding out that Person A had decided to allocate the chips him- or herself. Additionally, we explored their behavioral intentions. In Study 2, we did not investigate intentions, but actual behavioral reactions. However, Person B in the traditional TG has no behavioral option once Person A decides to distribute the outcomes him- or herself. To be able to study behavioral reactions, we therefore provided our participants with a behavioral option. After participants learned that A would allocate the outcomes him- or herself (by an active or inactive decision), participants were given the opportunity to divide a new number of scarce resources in a Dictator Game (DG; for an overview on this game, see for example Engel, 2011). In the DG that followed the TG, our participants allocated a new number of valuable resources (i.e., 75 chips, worth €0, 10 each) between themselves and a recipient. The recipient had no choice but to accept the proposed distribution.

We were interested in whether being denied the possibility to distribute outcomes in the TG would affect subsequent behavioral decisions. For this purpose, we informed half of our participants that the recipient in the DG was the same person (Person A) that they had been connected to in the TG. If, as Study 1 suggests, not being allowed to distribute outcomes is seen as an indication of low trust and leads to negative evaluations of the person held responsible (Person A), it seems plausible that at the behavioral level the participants would then allocate a relatively low number of chips to the recipient in the DG. In terms or reciprocity this could then be seen as a form of negative reciprocity (Gouldner, 1960; Fehr and Gächter, 2000; Falk and Fischbacher, 2006). Based on our findings of Study 1, we expected our participants to make lower allocations to the Person A in the DG if A’s prior decision to not let B distribute the outcome in the TG was active rather than inactive.

However, we also anticipated that the consequences of not being trusted might extend beyond the person being responsible for this decision. For this purpose, we also presented half of our participants with a DG in which they were not connected to Person A, but instead to a Person C, who had not in any way been involved in the previous TG setting. How would people allocate the outcomes with such an uninvolved Person C? Since C could not be held responsible for their fate in the TG, one might anticipate that they would not let their behavior with C be influenced by their prior experience. Research on generalized reciprocity (also called “paying it forward”) from Gray et al. (2014), however, indicates that negative reciprocity concerns may also affect encounters with uninvolved others.

Research on generalized reciprocity effects often focuses on situations with positive outcomes: A is kind to B, and as a consequence, B will be kind to C by paying the kindness forward (instead of only paying it backward to A). Insights from the study by Gray et al. (2014; also see Cardella et al., 2019) revealed that this effect also occurs in the negative domain. Their results revealed that participants who were confronted with a selfish decision in a previous interaction also became more selfish themselves in a subsequent interaction (as compared to how they behaved at baseline). For example, in their first experiment, participants received a selfish allocation ($0 out of $6) from an anonymous opponent in a DG. By subsequently asking these participants to interact with an entirely different person in another round of the DG, results revealed that the received negative outcome was paid forward: Participants proposed selfish offers to this new other person. However, they did not so if they first had received an equal ($3) or generous ($6) offer from their first opponent. Based on these findings, we thus expected that the negative experience of not being allowed to distribute outcomes in the TG, which was stronger for active than inactive decisions, might also be reflected in DG allocations to an uninvolved Person C (i.e., lower allocations in the active than in the inactive condition). However, we did expect lower allocations to Person A as compared to Person C (due to direct reciprocity).

## Materials and Methods

### Participants and Design

An a priori power analysis (using G^∗^Power3, Faul et al., 2007) was conducted to indicate our required sample size (for two-way ANOVAs fixed, special, main effects, and interactions, two-tailed; alpha = 0.05; power = 0.80). In total, a sample size of 128 was needed in order to detect a medium effect size (*f* = 0.25; Cohen, 1969). *Post hoc* analysis revealed that with our sample we obtained a power level of 0.80.

Participants in this study were 140 students (115 women, 25 men; *M* = 19.91 years, *SD* = 1.95) (see Text Footnote 1) from Leiden University who were paid 3 euros upon participation. The study had a 2 (type of decision: inactive vs. active) × 2 (allocation target: allocation to Person A vs. allocation to Person C) between-participants design. This study involving human participants was reviewed and approved by the Psychology Research Ethics Committee of Leiden University. The participants provided their written informed consent to participate in this study. The original data of Study 2 are publicly available and accessible via: https://osf.io/3jw6p/?view_only=bc512df8e91f4ac79d7a59f6f4bb61b8.

### Procedure

Unless reported differently, the design and procedure of Study 2 was identical to that of Study 1.

In the *inactive condition*, participants learned that Person A had received 20 chips (worth €0, 10 each) and had the following options: (Option 1) to divide the 20 chips him- or herself, or (Option 2) pass all chips to Person B (i.e., the participant) who would then divide the then tripled amount of 60 chips. In the *active condition*, Person B started out having 60 chips (worth €0, 10 each) and had the opportunity to divide this amount. Here Person A could: (Option 1) take this amount from Person B, the 60 chips would then be divided by three, and A was able to divide 20 chips him- or herself, or (Option 2) leave the initial 60 chips with Person B and therefore provide the participant with the opportunity to divide the 60 chips.

As in Study 1, participants learned, after a simulated decision time of 46 s, that A chose Option 1. But now they were also informed that A would immediately thereafter decide how to divide the 20 chips. After a simulated decision time of 34 s, participants learned that they (as Person B) had received an equal share from Person A (that is, 10 chips to B and 10 chips to A). This created a clear setting where it would be transparent to participants that they were (behaviorally) not trusted, but not (behaviorally) disadvantaged since A made an even distribution.

Subsequently, our first series of dependent measures was taken. Unless indicated differently, all items were measured on 7-point scales (1 = *absolutely disagree*; 7 = *absolutely agree*). The following measures were first assessed: Evaluations of Person A’s decision (e.g., ‘understandable’), experienced emotions (e.g., ‘frustrated’), and perceived main motives of A’s decision (e.g., ‘A did not want to take any risk’). See Tables 5–7 for all measured items per construct.

**TABLE 5:** Factor loadings based on an exploratory factor analysis with a principal axis factoring and direct oblimin rotation on 11 evaluation items (*N* = 140).

	1. General negative evaluation	2. Under- standable	Communality
6. Kind	−0.88		0.79
5. Unkind	0.83		0.74
11. Selfish	0.82		0.60
4. Social	−0.81		0.84
10. Cooperative	−0.74		0.57
8. Fair	−0.64		0.47
3. Antisocial	0.62		0.46
7. Unfair	0.60		0.41
9. Competitive	0.60		0.33
2. Incomprehensible		−0.91	0.82
1. Understandable		0.80	0.73
Percentage of variance	48.23	11.52	
Eigenvalue	5.70	1.53	
Cronbach’s α/Pearson’s *r*	0.91	0.77	

*Factor loadings < 0.30 are suppressed.*

**TABLE 6:** Factor loadings based on an exploratory factor analysis with a principal axis factoring and direct oblimin rotation on eight items measuring emotions (*N* = 140).

	1. Overall negative emotions	Communality
1. Satisfied	−0.86	0.61
5. Frustrated	0.80	0.61
2. Disappointed	0.79	0.55
4. Grateful	−0.72	0.44
3. Angry	0.67	0.62
8. Upset	0.65	0.60
6. Indignant	0.56	0.53
7. Surprised		0.15
Percentage of variance	49.42	
Eigenvalue	4.32	
Cronbach’s α	0.84	

*Factor loadings < 0.30 are suppressed.*

**TABLE 7:** Factor loadings based on an exploratory factor analysis with a principal axis factoring and direct oblimin rotation on nine items measuring the perceived underlying motives (*N* = 140).

	1. Risk/trust	2. Unfair-ness	3. Greed	Communality
3. A had lots of trust in me returning him/her a sufficient number of chips	0.76			0.60
1. A did not want to take any risk	−0.70			0.55
4. A distrusted me	−0.63			0.42
2. A wanted to take a risk	0.54			0.31
8. A wanted us together to possess as many chips as possible	0.49			0.28
9. A found that the chips had to be divided in a fair manner		−0.86		0.77
7. A wanted me to possess as many chips as A did		−0.42		0.20
6. Eventually, A wanted to possess more chips than me		0.87	0.74	
5. Eventually, A wanted to possess as many chips as possible for him- or herself		0.59	0.38	
Percentage of variance	22.28	16.84	8.12	
Eigenvalue	2.54	1.94	1.16	
Cronbach’s α/Pearson’s *r*	0.74	0.41	0.53	

*Factor loadings < 0.30 are suppressed.*

Next, participants filled out some questions to check whether the manipulation of *type of decision* (inactive vs. active) had been successful (e.g., ‘What was the starting point before A made his or her decision for Option 1? A possessed 20 chips; B possessed 60 chips’). Eight comprehension questions were asked to check whether participants had understood the main characteristics of the TG (e.g., ‘What was each chip worth?’; all between 97.9 and 100% correctly answered; for detailed results, see Supplementary Materials).

After all measures and checks were filled out, participants were introduced to the second phase of the study: A setting modeled after the DG that was used as a behavioral measure of not being trusted. All participants received a new number of chips (75 chips, worth €0, 05 each) and learned about the possibility to distribute these chips between themselves and another anonymous person. Participants in the allocation to Person A condition learned that the chips had to be allocated to the same person of the previous game (Person A), and participants in the allocation to Person C condition learned that the chips had to be allocated to an uninvolved, new participant (Person C). In the latter condition, participants additionally learned that Person A was going to be informed about the choice they had made concerning the allocation to Person C, so on that aspect conditions were identical. Again, full anonymity was ensured. As in Study 1, we only used neutral terms, and only referred to ‘Persons A, B, and C’ (e.g., we did not use labels like not ‘opponents’).

After the instructions of the DG, the following dependent measures were taken: Division of the 75 chips with A/C (i.e., sending 0 to 75 chips), and their main motives underlying the division (e.g., ‘I felt that the money had to be distributed in a fair manner’). See Table 8 for all measured items for this construct.

**TABLE 8:** Factor loadings based on an exploratory factor analysis with a principal axis factoring and direct oblimin rotation on 11 items measuring B’s main motives in the DG (*N* = 140).

	1. Fairness	2. Punishment	3. Trustworthiness	Communality
3. I wanted A to earn the same amount of money as I did	0.86			0.86
5. I found that the money had to be distributed in a fair manner	0.85			0.84
4. I wanted us together to earn as much money as possible	0.60			0.43
1. I wanted to earn as much money as possible	−0.47			0.26
2. I wanted to earn more money than Person A/C	−0.40			0.13
11. I wanted to show Person A that he/she took a wrong decision		0.95		0.84
7. I wanted to punish Person A		0.39		0.26
8. I wanted to show that I am a trustworthy person			1.02	0.98
9. I wanted to show to Person A that I am a trustworthy person			0.70	0.47
10. I felt morally obliged to give enough money to Person A/C			0.52	0.33
6. I wanted to help Person A			0.46	0.38
Percentage of variance	36.65	9.28	6.52	
Eigenvalue	4.39	1.33	1.22	
Cronbach’s α/Pearson’s *r*	0.79	0.40	0.78	

*Factor loadings < 0.30 are suppressed.*

Next, participants were asked some questions to check whether the manipulation of allocation target (allocation to Person A vs. allocation to Person C) had been successful (e.g., ‘Who were you distributing money to in this part? Person A; Person C)^2^. Six comprehension questions were administered to check whether participants had understood the main characteristics of the DG (e.g., ‘How many chips were available for division?’; all between 92.1 and 100% correctly answered).

Finally, participants were debriefed, thanked and paid.

## Results

### Manipulation Checks

In the *inactive condition*, all participants (100%) correctly answered that Person A started out receiving 20 chips in the TG. In the *active condition*, the majority (83.8%) correctly answered that not Person A, but Person B started out receiving 60 chips. And, regarding the subsequent DG, all participants (100%) in the *allocation to Person A* condition, and almost all participants (98.6%) in the *allocation to Person C* condition correctly identified their opponent. These findings suggest that our manipulations were perceived as intended.

### Exploratory Factor Analyses (EFAs)

Similar to Study 1, exploratory factor analyses (EFA) on each measured construct were conducted to explore the structure of the data (all KMOs = between 0.63 and 0.87, and all Bartlett’s tests of sphericity = *p*s < 0.001). All EFAs were conducted using principal-axis factor extraction and oblique rotation (direct oblimin rotation). See the Supplementary Materials for more details.

First, an EFA was conducted on the evaluations of Person A’s decision (eleven items) and narrowed this data down to two factors: ‘General negative evaluation’ (nine items; α = 0.91), and ‘understandable’ (two items; Pearson’s *r* = 0.77, *n* = 138, *p* < 0.001). Second, emotions following Person A’s decision (eight items) were narrowed down to one factor and one single item: ‘Overall negative emotions’ (seven items; α = 0.89), and the single item ‘surprised.’ Third, perceived main motives underlying Person A decision (nine items) were narrowed down to three factors: ‘Risk/trust’ (five items; α = 0.74), ‘unfairness’ (two items; Pearson’s *r* = 0.41, *n* = 138, *p* < 0.001), and ‘greed’ (two items; Pearson’s *r* = 0.53, *n* = 138, *p* < 0.001). See Tables 5–7 for all items per factor and corresponding factor loadings.

Person B’s main motives for the division in the DG (eleven items) were narrowed down to three factors: ‘Fairness’ (five items; α = 0.79), ‘punishment of A’ (two items; Pearson’s *r* = 0.40, *n* = 138, *p* < 0.001), and ‘trustworthiness’ (four items; α = 0.78). See Table 8 for all items per factor and corresponding factor loadings.

### Dependent Variables Phase 1

Phase 1 contained the TG interaction in which our participants were not trusted. Unless reported differently, the differences between the means of our two conditions (active vs. inactive) on the dependent variables were analyzed using separate one-way Analyses of Variance (ANOVAs).

#### Evaluations of Person A’s Decision

A significant effect of the type of decision was found on participants’ ‘general negative evaluation’ toward Person A’s decision, *F*(1,138) = 6.43, *p* = 0.01, $\eta_{\text{p}}^{2}$ = 0.05. Participants indicated a more negative evaluation toward A in the active condition (*M* = 2.43, *SD* = 1.26) than in the inactive condition (*M* = 1.94, *SD* = 1.04).

No significant difference was found on the ‘understandability’ of Person A’s decision between active (*M* = 5.06, *SD* = 1.69) and inactive (*M* = 5.13, *SD* = 1.81) decisions (*p* = 0.81).

#### Emotions

Self-reported ‘overall negative emotions’ following A’s decision were not rated as significantly different after active vs. inactive decisions (overall *M* = 2.21, *SD* = 1.24; *p* = 0.68). Likewise, no difference was found on the item ‘surprised’ (overall *M* = 4.32, *SD* = 1.84; *p* = 0.99).

#### Perceived Main Motives Underlying Person A’s Decision

Out of the three scales capturing the perceived main motives, ‘unfairness’ was perceived as significantly more important for A’s decision by participants in the active condition (*M* = 2.37, *SD* = 1.35) than in the inactive condition (*M* = 1.86, *SD* = 1.01), *F*(1,138) = 6.37, *p* = 0.01, $\eta_{\text{p}}^{2}$ = 0.04.

No significant differences were observed for ‘risk/trust’ (overall *M* = 2.62, *SD* = 1.25; *p* = 0.61) and ‘greed’ (overall *M* = 1.81, *SD* = 1.10; *p* = 0.51).

### Dependent Variables Phase 2

Phase 2 contained participants’ allocation of the 75 chips in the DG.

#### DG Allocation to Person A/C

A 2 (type of decision: active vs. inactive) × 2 (allocation target: allocation to Person A vs. allocation to Person C) ANOVA on participants’ allocation in the DG revealed a significant main effect of type of decision, *F*(1,136) = 4.64, *p* = 0.03, $\eta_{\text{p}}^{2}$ = 0.03. Participants in the active condition (*M* = 32.18, *SD* = 11.00) allocated significantly fewer chips to their opponent as compared participants in the inactive condition (*M* = 36.14, *SD* = 10.49).

No main effect of allocation target was found on the allocation to Person A (*M* = 33.25, *SD* = 10.63) and Person C (*M* = 35.15, *SD* = 11.11, *p* = 0.32). Also, no interaction effect was found (*p* = 0.48).

#### Participants’ Main Motives in the DG

Only a marginal main effect of type of decision on the main motive ‘punishment of A’ was found, *F*(1,136) = 3.37, *p* = 0.07, $\eta_{\text{p}}^{2}$ = 0.02. Actively not trusted participants rated this motive as more applicable (*M* = 2.07, *SD* = 1.34) than inactively not trusted participants (*M* = 1.69, *SD* = 1.10). No significant main effect of allocation target (overall *M* = 1.87, *SD* = 1.23; *p* = 0.46) or interaction effect (*p* = 0.24) was found.

No main effects or interaction effects were found for the motives ‘fairness’ (overall *M* = 4.86, *SD* = 1.51) and ‘trustworthiness’ (overall *M* = 5.19, *SD* = 1.35; all *p*s between 0.24 and 0.88).

## Discussion

In Study 2 we again manipulated whether Person A had made an active vs. inactive decision to not let the participant distribute the outcomes in the TG. By adding a subsequent DG, we investigated whether the effects of not being trusted would go beyond affective responses (Study 1). Moreover, we also investigated whether behavioral reactions would be restricted to the person who had decided to distribute the outcomes him- or herself (Person A) or whether these reactions would also extend to an uninvolved interaction partner (Person C).

Similar to Study 1, results of Study 2 underlined the importance of distinguishing between action and inaction. Study 2 revealed that active versus inactive decisions also had an impact at a behavioral level. Specifically, participants allocated fewer chips to the recipient in the DG when in the prior TG Person A had actively denied them the opportunity the distribute outcomes than when this was the result of an inactive decision. Remarkably, this behavioral effect not only occurred when participants faced the person responsible (Person A, which may be explained by direct negative reciprocity) but also when facing an uninvolved other (Person C, which may be explained by generalized negative reciprocity).

It is also noteworthy that we observed these effects in a setting where participants, in contrast to Study 1, learned that although Person A had decided to allocate the chips him- or herself, Person A had subsequently decided to distribute the outcomes evenly between A and B. In other words, participants had learned that Person A had not benefited him- or herself at their expense. Even under these circumstances, we found that at the behavioral level it mattered whether A’s decision to not let B allocate the chips had been an active or inactive decision. The effect was also strong enough to affect the evaluations of A, and his/her perceived motives, although it did not emerge in the self-reported emotions.

## General Discussion

In two studies, we assessed the consequences of not being trusted. In our paradigm, Person B received information about the other Person A’s decision, but received no explicit statements that their opponent had not trusted them. Also, participants were not informed about the underlying motive behind Person A’s decision. This setting matches many real-life encounters in which people have little more than what others do (or not do) to rely on. But of course, people will interpret other people’s behavior, and react according to the inferences they make.

For the current purposes, it is important to note that the findings obtained in Study 1 showed that the participants indeed perceived Person A’s decision as an indication of low trust. This fits with the findings of previous research that decisions in trust games are indicative of trust, and for example cannot be reduced to mere risk decisions (e.g., Bohnet and Zeckhauser, 2004) or self-interested motives (see for neurological evidence Declerck et al., 2013). In our studies A’s decision not to let B distribute the chips was not necessarily related to self-interest because A could (and did) divide to outcomes evenly. In this respect, the set-up of our trust game was different from most trust games (but not all, see e.g., Van Dijk et al., 2017), in which the distribution of outcomes if A chooses Option 1 is fixed, and in the advantage of A. This setup allowed us to isolate the decision not to trust from self-benefiting behavior.

While in Study 1, participants could (and did) anticipate an unequal distribution, in Study 2 they thus immediately learned that A had distributed the outcomes equally. This difference might also explain why, in Study 2, we did not find an effect of active vs. inactive trust on participants’ self-reported emotions or perceptions of (un)fairness. The fact that we did, however, find an effect on giving in the dictator game is related to perceptions of not being trusted. At the same time, we do not want to claim that the negative reactions to the Person A’s behavior should solely be attributed to trust inferences. Indeed, it should also be noted that, by not trusting the participant, A lowered the collective outcomes. As research on social dilemmas has repeatedly shown, people may react negatively to this aspect as well (for an overview on social dilemmas, see Van Lange et al., 2013; for a more focused review on how people react to reduction of collective outcomes, see Van Dijk et al., 2008).

Our studies combined show that addressing reactions to not being trusted may generate relevant new insights. In the current paper we revealed both the affective and behavioral consequences of not being trusted, and demonstrated that these may be more impactful after active than after inactive decisions. With this the findings also extend previous insights on inaction-action effects (Kahneman and Tversky, 1982; Kahneman and Miller, 1986; Ritov and Baron, 1990, 1992; Spranca et al., 1991) by showing that these effects are also relevant in the domain of interpersonal trust. Active decisions (i.e., withholding someone of the opportunity to prove him- or herself trustworthy) cause stronger negative effects as compared to inactive decisions (i.e., not providing someone the opportunity to prove him- or herself trustworthy). Moreover, the results revealed that the negative behavioral consequences may even extend to uninvolved others (i.e., Person C in Study 2). As we argued above, this finding is informative for the literature on reciprocity in the sense that it suggests that not being trusted may induce both direct negative reciprocity (cf. Gouldner, 1960; Fehr and Gächter, 2000; Falk and Fischbacher, 2006) and generalized negative reciprocity (cf. Keysar et al., 2008; Rankin and Taborsky, 2009; Gray et al., 2014).

While our findings connect in a meaningful way to other research, it is important not to overstate our findings and conclusions. In this respect, it is also good to discuss the magnitude of our effects. For example, the effect of active vs. inactive trust on the subsequent allocation in the dictator game was significant, but we acknowledge that the effect size was small. This does not make the effect unimportant, nor can it be attributed to an unusually large sample size; our study was sufficiently powered but not overpowered. Nevertheless, we feel that future research may be useful to test the robustness of this specific finding, and in doing so maybe combine this with assessing the effects of decision type on emotions and evaluations. When focused on the behavioral aftermath of not being trusted, such research may also be used to follow-up on an observation of one of the reviewers of this article, namely, that in Study 2 we observed similar behavioral reactions to Person A and Person C while one might expect that allocations would be lowest to the person responsible for the distrust (i.e., person A). First of all, one might wonder whether the fact that allocations were not different for A and C could be explained by a floor effect that kept people from lowering their allocations to A. Since allocations to A were not extremely low (i.e., participants still allocated 33.25 of the 75 chips to A), we consider this explanation unlikely. Relatedly, we do not feel that null effects in the studies may be attributed to low sample sizes, given that we our studies were sufficiently powered. An alternative account, however, would be that the underlying motives differed for allocations to A and C. One could argue that the behavioral effects are due to both direct negative reciprocity (to A) and generalized, negative reciprocity (to C). However, it should be noted that contributions to C are not necessarily an indication of generalized reciprocity. After all, when connected to Person C, participants learned that their subsequent allocation would also become known to the player A, i.e., the person who had not trusted them. This aspect could have provided an additional motive to participants; e.g., they might have taken the opportunity to signal to Player A that they felt his/her decision not to trust them was not just, or to make player A feel bad. Note, however, that one could also formulate a different argument, namely, that participants could have taken the opportunity to make a fair allocation to thereby communicate to Person A that they would have been a trustworthy partner. That they did not do so, suggests that sending a punitive signal to A may indeed have been important to them. In any case, the fact that our setup would have allowed for such additional motives to come into play indicates that future research could also study the aftermath in settings in which one’s decision would not become known to player A. This would further contribute to the field of generalized/indirect reciprocity, and provide some new insights regarding the mixed findings regarding the existence of generalized reciprocity in dictator games (see Bartlett and DeSteno, 2006; Capraro and Marcelletti, 2014).

Within the field of social decision-making it is well-acknowledged that decisions, including dictator game decisions, can be influenced by people’s emotions (e.g., Capraro, 2019). A punitive signal can, but does need not be, emotion-driven. The fact that in Study 2, we did not observe high levels of negative emotions, which were also not different between active and inactive decisions, suggests that emotions may not have been the primary driver. Such an interpretation would fit with previous theorizing as well as empirical research on sanctions. For example, in their study on timing effects and sanctions, Molenmaker et al. (2019) concluded (and observed) that sanctioning does not necessarily have to be attributed to ‘heated tempers.’ In a similar vein, in their review on justice in social dilemmas, Schroeder et al. (2003) distinguished between retribution that is more emotionally-driven and impulsive, and retribution that is more calculative and instrumental. Based on the current findings we tentatively suggest that in the current setup, sending the signal to A via allocations in a subsequent dictator game may have been more calculative than emotion-driven. A possible calculative reason could, for example, be that participants wanted to deter A from engaging in future acts of no trust rather than to directly retaliate. We realize that this is a *post hoc* explanation, but it can be put to the test in future research.

Future studies could also be used study whether not being trusted causes persons to trust others less as well. Previous research revealed that after unreciprocated trust, people in return place less trust in others in subsequent interactions (Erev and Roth, 1998; Camerer and Ho, 1999). In a similar vein, one might study whether distrust breeds distrust. As Juvina et al. (2013) showed, carry-over effects between games may be stronger when games are more similar. If this would translate to the current findings, carry-over effects after not being trusted may be more pronounced if subsequent decisions are also about trust. If so, effects might then be stronger than we currently found in our Study 2 (where the subsequent decision was a dictator game that did not involve trust).

Another question for future research is whether the effects of action and inaction would also hold for the act of providing trust. In the traditional TG, placing trust in another means that Person A actively sends the money to Person B. If this active type of trust would cause more positive consequences as compared to an inactive type of trust, the high rates of trustworthiness that are often found may be (partly) due to the taken action by the trustor. Studying these differences in types of trust would eventually teach researchers more about what exactly is studied using the TG paradigm. And as we know from the omission/commission literature, this significant distinction in its effects and responses is something to take into account.

### Limitations and Practical Implications

Economic games, such as the trust game, are often-used paradigms to study decision-making processes in social interactions. The elegance of these paradigms is that they are not only easy to use and understand for participants due to their simplicity, but also that their research outcomes are easily comparable to results from distinct studies using similar paradigms [see, for example, Johnson and Mislin (2011) for a meta-analysis with data aggregated from 162 studies using trust game paradigms]. These advantages, also come with limitations. For instance, due to their simplicity and abstractness, findings from such economic games might not directly translate to more complex real-world settings. It would therefore be good for future research to investigate whether these findings can be replicated in real world trust settings with more complexity (e.g., the online trading example we provided in our introduction), and use these studies to also study populations other than the student samples used in our current experiments. Research along these lines can further inform us on the generalizability and robustness of the findings. If such studies would corroborate the current findings, one of the more practical implications could be that people should especially be careful of sending signals that might communicate low levels of trust as it may not only be negatively evaluated, but also lead to upstream negative behavioral effects.

### Conclusion

It has been acknowledged that not placing trust in another (in an economic game setting) may be costly (Fetchenhauer and Dunning, 2009). People may miss out on potential gains because of undue skepticism about the opponent’s level of trustworthiness. Reciprocated trust could provide extra earnings that will be missed by those not enabling others to act trustworthy. Our studies indicate that the aftermath of no trust may be even more costly than missing out on potential positive outcomes. Not trusting others has direct negative affective consequences for those who are not trusted, and can lead to lower outcomes in subsequent encounters. This paper therefore contributes to the growing literature concerning low trust and its multilevel consequences, by revealing that the detrimental aftermath of (especially active) decisions reaches further than the trust situation itself, and not just holds for the initial parties involved.

## Data Availability Statement

The original data of Study 1 and 2 are publicly available and accessible via: https://osf.io/3jw6p/?view_only=bc512df8e91f4ac79d7a59f6f4bb61b8.

## Ethics Statement

The studies involving human participants were reviewed and approved by Psychology Research Ethics Committee of Leiden University. The patients/participants provided their written informed consent to participate in this study.

## Author Contributions

MS, ED, and EK contributed to the conception and design of the studies. MS and ED organized the data collection. MS performed the statistical analyses. MS, ED, EK, and WD were involved in the interpretation of the data and process. All authors helped writing the final version of this manuscript and read and approved the submitted version.

## Conflict of Interest

The authors declare that the research was conducted in the absence of any commercial or financial relationships that could be construed as a potential conflict of interest.

## Footnotes

1 As we had more women than men in our sample, and earlier research has shown that women tend to donate slightly more than men in dictator games (for an overview, see Engel, 2011), we also analyzed whether men and women showed different behaviors in Study 2. Specifically, we included gender as a covariate in our ANOVA analysis of giving behavior. This analysis showed that gender had no significant effect on giving, *F*(1,138) = 0.04, *p* = 0.85, $\eta_{\text{p}}^{2}$ = 0.00 (*M*_men_ = 33.84, *SD* = 16.79, and *M*_women_ = 34.30, *SD* = 9.22). Moreover, adding gender as a covariate did not change the effects of our manipulations.

2 In our studies we did not exclude participants who failed the manipulation check(s) (see also Aronow et al., 2019). The results of our analyses with and without exclusion of these participants were similar for both experiments (with similar means and standard deviations). Excluding participants who failed the checks in Study 2 would the make effect of inactive vs. active trust on allocations in the DG marginally significant (*p* = 0.09), which is most likely due to the reduction in power.
