# Supplementary material for: The Detrimental Effects of No Trust: Active Decisions of No Trust Cause Stronger Affective and Behavioral Reactions Than Inactive Decisions
Source: Front Psychol. 2021 Jul 7;12:643174. doi: 10.3389/fpsyg.2021.643174 (PMC8293391; doi:10.3389/fpsyg.2021.643174)
Supplement: Supplementary file 1 [file Data_Sheet_1.pdf]

Supplementary Material

The Detrimental Effects of No Trust: Active Decisions of No Trust Cause Stronger Affective and Behavioral Reactions than Inactive Decisions

Schutter, M., Van Dijk, E., De Kwaadsteniet E.W., & Van Dijk, W.W.

In this section, additional statistics of the main results of Study 1 and 2 are reported. We also report the exploratory measures of Study 1 (see the Materials and Methods section in the main article). All measured items of both studies are reported either here or in the main article.

1 Study 1: Affective Reactions Towards a No Trust Decision

1.1 Comprehension questions

Results show that participants understood the main features of the TG. Participants correctly identified the value per chip (100%, assessed twice: before and after the TG), their role as Person B within the game (99.2%), the anonymity of their role and decisions (100%), that the final outcome would eventually be paid (95.2%), and the consequences of opting for Option 1 (100%) and Option 2 (100%).

1.2 Factor Analyses of the dependent measures

First of all, evaluations of Person A's decision to opt for Option 1 (i.e., to divide the money by A him- or herself) were assessed by twelve items. This sample of data appeared suitable for factoring, as indicated by the high strength of the relationships among variables (Kaiser-Meyer Olkin, KMO=.85, above the recommended .60), and a significant Bartlett's test of sphericity ($\chi 2$ [66] = 782.30, $p<.001$). EFA narrowed this sample this down to two factors. Factor 1 was comprised of nine items that explained 37.22% of the variance (with factor loadings ranging from .50 to .80). We labeled this factor 'general negative evaluation' (Cronbach's α = .87). Factor 2 was comprised of three items that explained 16.80% of the variance (with factor loadings ranging from .97 to .85). We labeled this factor 'perceived expectedness' (α = .93).

Secondly, (self-reported) emotions following Person A's decision were assessed by fifteen items. This sample of data appeared suitable for factoring, as indicated by the high strength of the relationships among variables (KMO=.82), and a significant Bartlett's test of sphericity ($\chi 2$ [105] = 841.37, $p<.001$). EFA narrowed this sample this down to three factors. Factor 1 was comprised of seven items that explained 32.32 % of the variance (with factor loadings ranging from .41 to .78). We labeled this factor 'negative emotions' (α = .87). Factor 2 was comprised of five items that explained 15.02% of the variance (with factor loadings ranging from .60 to .81). We labeled this factor 'positive emotions' (α = .82). Factor 3 was comprised of three items that explained 4.55% of the variance (with factor loadings ranging from .43 to .68). We labeled this factor 'personal rejection' (α = .67).

Thirdly, the perceived main motives underlying Person A decision were assessed by eight items. This sample of data appeared suitable for factoring, as indicated by the high strength of the relationships among variables (KMO=.71), and a significant Bartlett's test of sphericity ($\chi 2$ [28] = 252.23, p<.001). Based on the Kaiser criterion of eigenvalues ≥ 1.0 (which we used up to now), the data would be reduced to three factors. However, based on the scree plot and interpretation of content, this sample was narrowed down to two factors. Factor 1 was comprised of six items that explained 32.60% of the variance (with factor loadings ranging from .43 to .74). We labeled this factor 'low trust/risk' (α = .69). Factor 2 was comprised of two items that explained 9.83% of the variance (with factor loadings ranging from .75 to .79). We labeled this factor 'control' (Pearson's *r* [122] = .57, $p <$ .001).

1.3 Factor analysis of the additional measure

Behavioral intentions towards Person A were assessed by six items derived from the Transgression-Related Interpersonal Motivations Scale (TRIM). This sample of data appeared suitable for factoring, as indicated by the high strength of the relationships among variables (KMO=.83), and a significant Bartlett's test of sphericity ($\chi 2$ [15] = 295.46, p<.001). Based on interpretation of content, this sample was narrowed down to one factor. Factor 1 was comprised of five items that explained 48.89% of the variance (with factor loadings ranging from .53 to .65). We labeled this factor 'negative behavioral intention' (α = .85). Only the item 'I want to prove that I am trustworthy' did not load sufficiently high on this factor (factor loading <.30) and was therefore analyzed separately in further analyses.

1.4 Exploratory measures

1.4.1 Satisfaction with the outcome

Participants' satisfaction with the final allocation was assessed using the items: 'How satisfied are you with the final outcome?', and 'How satisfied are you with the 10 chips?'. These items were thus measured *after* people found out that Person A allocated the available money equally.

ANOVAs were conducted to assess the differences between an active and inactive decision on the two items. First, a significant main effect was found of the type of decision (active vs. inactive) on the satisfaction with the final outcome, $F(1, 122) = 4.33$, $p = .04$, $\eta_p^2 = .03$. Participants in the action condition ($M = 5.19$, $SD = 1.50$) were less satisfied with the final outcome compared to participants in the inactive condition ($M = 5.71$, $SD = 1.25$).

After an active decision, participants ($M = 5.21$, $SD = 1.46$) were equally satisfied with the received number of chips (i.e., 10 chips) as compared to after an inactive decision ($M = 5.56$, $SD = 1.30$; $p =$ .16, $\eta_p^2 = .02$).

2 Study 2: Affective and Behavioral Reactions to a No Trust Decision

2.1 Comprehension questions

Our results show that participants understood the main features of the TG. They correctly identified their role to be Person B within the game (97.9%), the anonymity of their role/decisions (98.6%), the consequences of opting for Option 1 (98.6%) and Option 2 (98.6%), the value of €0,10 per chip (100%), and how Person A eventually distributed the 20 chips (i.e., equal; 100%).

Furthermore, participants also correctly identified the main features of the DG. They correctly identified their role as Person B within the game (97.1%), the anonymity of their role/decisions (98.6%), the number of chips available for allocation (100%), the value per chip (99.3%), and the fact that Person A would always be made aware of the allocation they made (92.1%).

2.2 Factor analyses of the dependent measures, phase 1 (TG)

First of all, evaluations of Person A's decision were assessed by eleven items. This sample of data appeared suitable for factoring, as indicated by the high strength of the relationships among variables (Kaiser-Meyer Olkin, KMO=.84, above the recommended .60), and a significant Bartlett's test of sphericity ($\chi 2$ [55] = 1019.61, p<.001). EFA narrowed this sample this down to two factors. Factor 1 was comprised of nine items that explained 48.23% of the variance (with factor loadings ranging from .62 to .82). We labeled this factor 'general negative evaluation' (α = .91). Factor 2 was comprised of two items that explained 11.52% of the variance (with factor loadings ranging from .80 to .91). We labeled this factor 'understandable' (Pearson's r [138] = .77, p < .001).

Secondly, (self-reported) emotions following Person A's decision were assessed by eight items. This sample of data appeared suitable for factoring, as indicated by the high strength of the relationships among variables (KMO=.87), and a significant Bartlett's test of sphericity ($\chi 2$ [28] = 550.18, p<.001). Based on interpretation of content, this sample was narrowed down to one factor and one single item. Factor 1 was comprised of seven items that explained 49.42% of the variance (with factor loadings ranging from .56 to .86). We labeled this factor 'overall negative emotions' (α = .89). The item 'surprised' did not load sufficiently high on Factor 1 and was therefore analyzed separately in further analyses.

And thirdly, 'perceived main motives' underlying Person A decision were assessed by nine items. This sample of data appeared suitable for factoring, as indicated by the high strength of the relationships among variables (KMO=.63), and a significant Bartlett's test of sphericity ($\chi 2$ [63] = 288.75, p<.001). EFA narrowed this sample this down to three factors. Factor 1 was comprised of five items that explained 22.23% of the variance (with factor loadings ranging from .49 to .76). We labeled this factor 'risk/trust' (α = .74). Factor 2 was comprised of two items that explained 16.84% of the variance (with factor loadings ranging from .42 to .86). We labeled this factor 'unfairness' (Pearson's r [138] = .41, p < .001). Factor 3 was comprised of two items that explained 8.12% of the variance (with factor loadings ranging from .59 to .87). We labeled this factor 'greed' (Pearson's r [138] = .53, p < .001).

2.3 Factor analyses of the dependent measures, phase 2 (DG)

The 'perceived main motives' underlying Person B's decision in the DG were assessed by eleven items. This sample of data appeared suitable for factoring, as indicated by the high strength of the relationships among variables (KMO=.79), and a significant Bartlett's test of sphericity ($\chi 2$ [55] = 639.47, p<.001). EFA narrowed this sample this down to three factors. Factor 1 was comprised of five items that explained 36.65% of the variance (with factor loadings ranging from .40 to .86). We labeled this factor 'fairness' (α = .79). Factor 2 was comprised of two items that explained 9.28% of the variance (with factor loadings ranging from .39 to .95). We labeled this factor 'punishment of A' (Pearson's r [138] = .40, p < .001). Factor 3 was comprised of four items that explained 6.52% of the variance (with factor loadings ranging from .46 to 1.02). We labeled this factor 'trustworthiness' (α = .78).
